# Hormone-Balancing Effect of Pre-Gelatinized Organic Maca (Lepidium peruvianum Chacon): (III) Clinical responses of early-postmenopausal women to Maca in double blind, randomized, Placebo-controlled, crossover configuration, outpatient study

**Published:** 2006-12

**Authors:** H. O. Meissner, A. Mscisz, H. Reich-Bilinska, P. Mrozikiewicz, T. Bobkiewicz-Kozlowska, B. Kedzia, A. Lowicka, I. Barchia

**Affiliations:** 1 *Faculty of Health Studies, Charles Sturt University & Therapeutic Research International, GPO Box 4792, Sydney 2001, Australia;*; 2 *Research Institute of Medicinal Plants, 27 Libelta St., 61-707 Poznan, Poland; 18, 07743 Jena;*; 3 *Specialist Gynecology Private Clinic, Glogow, Poland;*; 4 *Department of Pharmacology, Medical University, Poznan, Poland;*; 5 *Department of Primary Industry, E. Macarthur Institute, Menangle, Australia*

**Keywords:** blood biochemistry, early-postmenopause, hormones, HRT, maca, menopausal symptoms

## Abstract

This is the second, conclusive part of the clinical study on clinical responses of early-postmenopausal women to standardized doses of pre-Gelatinized Organic Maca (Maca-GO). Total of 34 Caucasian women volunteers participated in a double-blind, randomized, four months outpatient crossover configuration Trial. After fulfilling the criteria of being early-postmenopausal: blood Estrogen (E2<40 pg/ml) and Follicle Stimulating Hormone (FSH>30 IU/ml) at admission, they were randomly allocated to Placebo (P) and Maca-GO (M) treatments (2 groups of 11 participants each). Two 500 mg vegetable hard gel capsules with Maca-GO or Placebo powder were self-administered twice daily with meals (total 2 g/day). At admission and follow-up monthly intervals, body mass index (BMI), blood pressure, levels of gonadal, pituitary, thyroid and adrenal hormones, lipids and key minerals were measured. Bone markers were determined after four months M and P use in 12 participants. Menopausal symptoms were assessed according to Greene’s Score (GMS) and Kupperman’s Index (KMI). Data were analyzed using multivariate technique on blocs of monthly. Results and canonical variate technique was applied to GMS and KMI matrices. Two months application of Maca-GO stimulated (*P*<0.05) production of E2, suppressed (*P*<0.05) blood FSH, Thyroid (T3) and Adrenocorticotropic hormones, Cortisol, and BMI, increased (*P*<0.05) low density lipoproteins, blood Iron and alleviated (*P*<0.001) menopausal symptoms. Maca-GO noticeably increased bone density markers. In conclusion, Maca-GO applied to early-postmenopausal women (i) acted as a toner of hormonal processes along the Hypothalamus-Pituitary-Ovarian axis, (ii) balanced hormone levels and (iii) relieved symptoms of menopausal discomfort, (hot flushes and night sweating in particular), thus, (iv) exhibited a distinctive function peculiar to adaptogens, providing an alternative non-hormonal plant option to reduce dependence on hormone therapy programs (HRT).

## INTRODUCTION

In the previous paper from this series (1), the clinical effects of administering a preparation of the non-hormonal root of the Andean plant *Lepidium peruvianum* Chacon - pre-Gelatinized Organic Maca (Maca-GO) were studied on 124 early-postmenopausal women. It has been concluded that the non-hormonal Maca-GO preparation exhibits a hormone-balancing effect on the female organism and thus reduces menopausal discomfort. Those results confirmed earlier short- and long pilot clinical studies (2). However, discrepancies existed between results obtained in both the previous (1) and pilot study on women (2) and in laboratory models on ovariectomised rats (3), mainly in responses of estrogen (E2), follicle stimulating hormone (FSH), progesterone (PRG) luteinizing hormone (LH), lipid metabolism indicators (triglycerides -TRGL; cholesterol -CHOL; high- and low density lipoproteins - HDL and LDL) and minerals (Calcium - Ca; Phosphorus - P; and Iron - Fe) to Maca-GO treatment. Also, there were inconsistencies in the responses of rats to short- and long-term administration of Maca-GO in concentration of minerals in bone and muscle tissues relative to mineral metabolism indicators based on blood mineral profiles (4). Therefore, in this, second and concluding part of the clinical study which is a continuation of the previously-reported research (1), physiological and symptomatic responses of early-postmenopausal women to standardized doses of Maca-GO were investigated in relation to changes which may exist along a pituitary, thyroid, adrenal and gonadal axis, as measured by concentrations of relevant hormones. Simultaneously, serum lipids and key minerals were also analysed.

In this double blind, randomized, outpatient, four months crossover design clinical Trial (2 × 2 months), the effect of two months Maca-GO, parallel to Placebo treatment on early-postmenopausal women, was studied. In addition to analysis of four previously studied (1) hormones (FSH, E2, PRG and LH), blood was analyzed for Cortisol (CT), Adrenocorticotropic hormone (ACTH), Thyroid hormones (thyroid stimulating hormone - TSH, thyroxine - T3 and T4) and lipids (TRGL, CHOL, FSH and LDL), parallel to observations of bone density markers. Using canonical variate analysis, statistical ranking of individual menopausal symptoms contributing to severity of overall discomfort felt by menopausal women was made as subjectively assessed by responses of participants to menopausal tests according to Greene’s score (MGS) and Kupperman’s index (MKI).

## MATERIAL AND METHODS

### Maca

The Detailed description of Maca root (*Lepidium peruvianum* Chacon), its botanical characteristics, traditional and current applications were described in details by Chacon (5) and Obregon (6) and Maca-GO preparation used in this Trial was obtained from the same batch of the product described in detail previously (1, 3).

### Subjects

All subjects considered for inclusion in the study were early-postmenopausal women in good overall health and fulfilling menopausal criteria set at the levels: for follicle-stimulating hormone (FSH) of 30 IU/mL level or more and E2 of 40 pg/mL levels or less. Subjects had experienced absence of menses for at least 6 months, were not on HRT, or had discontinued HRT at least 12 months before admission to the study. Subjects were excluded if they had a history of breast cancer, hyperplasia, endometrial carcinoma, or cervical neoplasia; undiagnosed abnormal vaginal bleeding; a bilateral hysterectomy; history of cardiovascular disease; liver disease; history of chronic alcoholism, medication hypersensitivity, or allergy to dietary supplement ingredients; uncontrolled addiction or severe depression; acute systemic infection or abnormal laboratory values.

The study was conducted on early-postmenopausal women selected from patients regularly visiting a gynecologic clinic CG-1 in Glogow municipality, the demographics of which were given previously (1).

As a result of screening procedures, a total of 34 participants representing Caucasian, early-postmenopausal women volunteers aged 49 to 58 years were selected. They represented healthy subjects willing to participate in the study of four months duration. The protocol in the crossover Trial required five visits to the clinic: screening as a baseline at an admission visit (A) and four, monthly, follow-up visits. In the pilot bone density observation, two visits were required: at admission and after four months of either Placebo (P) or Maca-GO (M) treatment.

Two groups of 11 subjects each were used in a Trial according to a crossover design and the following randomized treatment sequence allocation: A-P-P-M-M and A-M-M-P-P. Two groups of 6 women (M and P) were involved in a forearm bone density observation, together with levels of blood FSH and E2 recorded at the admission point and at the time of bone density scanning four months later.

### Procedure

The protocol and amendments to the entire study (1) were approved by the Bioethics Committee of Medical Review Board in Poznan (No. 11/2004). Written informed consent was obtained from each enrolled subject regarding voluntary participation in the trial conducted under specialist Gynecologist’s supervision and after explanation of the purpose, benefits and possible risks of the study, its requirements and procedures.

The Gynecologist-investigator enrolled all patients and randomly allocated subjects to experimental groups, after previous clinical confirmation of blood FSH and E2 eligibility criteria. The patients as well as the research team were kept blind throughout the study.

At the admission and during further consultation visits to the gynecologist, each eligible subject was given a complete physical and pelvic examination, blood was sampled for clinical tests and an assessment of specific symptoms describing menopausal status was made during an interview by the doctor using the questionnaires according to GMS and KMI. Subjects were instructed to return any unused portion of the monthly volume of capsules, so as to determine compliance.

### Experimental protocol

The protocol was identical to the one applied in the previous part of the study (1). At the start and then on completion of each monthly interval of the study, all women were interviewed by the gynecologist and requested to answer a set of standard questions according to questionnaires designed by Greene (menopausal score) and Kupperman (menopausal index). At the same time, body weight and blood pressure were checked and blood was sampled for hormones and other biochemical analyses.

The study was carried out by specialist Gynecologist and researchers of the Research Institute of Medicinal Plants and Medical University in Poznan under the strict supervision of the Study Coordinator between January 2004 and June 2005.

### Assays

Blood serum levels of hormones were measured on a monthly basis: 17β-estradiol (E2), FSH, LH and PRG as well as, CT, ACTH, and TSH, T3, T4). Blood pressure, body weight, serum mineral contents (Ca, P, Fe) and lipid profiles (CHOL, TRGL, HDL, LDL) were also determined at monthly intervals together with indices of menopausal discomfort determined in personal interviews by doctor with the use of questionnaires of Greene (2) and Kupperman accordingly (7).

Blood analyses were conducted by “Prodok” Medical Laboratory, Przylep, Poland, using the officially accepted standard clinical procedures on Immulite - DPC equipment. Precision of analytical techniques in both Laboratories is monitored by National Center of Quality of Diagnostic Medical Laboratories in Poland and both Laboratories are participants in the International Quality Control RIQAS maintained by Randox Company.

Bone density was measured by applying standard diagnostic system (equipment and technique) adopted in public hospital (Diagnostic & Screening Department, Copper Industry Health Centre, Lubin, Poland) for routine bone density assessment according to National Screening Program principles. Bone density of forearm was conducted on STRATEC XCT-960 PQCT equipment.

### Statistical analysis

Data were expressed as means with a linear mixed model fitted to data, allowing the comparison between groups of individuals within each month and taking into account the effects of individuals on each response variable (combined fixed and random effect). The errors were assumed to follow a Gaussian (normal) distribution. All parameters were estimated using the Restricted Maximum Likelihood (REML) estimation (8). The comparisons between groups within months were made using least significant difference (LSD) test with the differences considered significant at *P*<0.05 and highly significant at *P*<0.01 and *P*<0.001 level.

**Greene’s and Kupperman’s scores of post-menopausal symptoms:** Analysis of total scores defined by both Greene (GMS) and Kupperman (KMI) were analyzed using the conventional analysis of variance to compare the individual groups (Menopausal status by Maca treatment combinations). Weighted total scores of 21 in GMS and indexed 11 symptoms based on KMI formula were analyzed in a similar way to the hormone data. Significant differences were tested using the least significant difference (LSD) at 5% level.

A multivariate analysis via the canonical variate technique (9) was used to relate 21 (GMS) or 11 (KMI) response variates to groups of menopausal women treated in various sequence combination of Maca & Placebo treatment. Canonical variate coefficients (Vector loadings) were calculated from the sums of squares and product matrix of the 21 GMS or 11 KMI response variates and corresponding canonical variate scores were calculated from the first two largest eigen-values. Monthly data were analyzed separately. All analyses were performed using SAS statistical software (10).

## RESULTS

### Subjects’ participation

The study commenced with total of 34 subjects registered at the admission point: 22 in a crossover configuration Trial and 12 in a Pilot Bone Density Observation.

All 22 women participating in the crossover Trial completed four months of the study. In the Pilot Bone Density Observation, two participants from Placebo group and one from Maca-GO group have not completed the four months study as they failed to take their daily doses of capsules and therefore, they were excluded from the analysis of data.

Table 1 summarizes data on BMI, systolic and diastolic blood pressure of participants during this 4-month crossover Trial. Maca-GO treatment significantly (*P*<0.05) reduced BMI in the sequence group in which Placebo was introduced for two months prior to two months of Maca-GO treatment (APPMM). No significant (*P*>0.05) effect was recorded in either systolic or diastolic blood pressure in relation to admission point nor to Placebo treatment, although, Maca-GO showed a slight tendency (*P*>0.05) to lower systolic blood pressure.

**Table 1:** Average Admission (A) and monthly values for Body Mass Index (BMI), Systolic (SBP) and Diastolic (DBP) Blood Pressure at five sampling points (Month Model)^a^ and Placebo (P) versus Maca-GO (M) contrast (Treatment Model) for two application sequences in a crossover design: A[PP × MM] and A[MM × PP] (n=11 in both groups) during four months long study

Measurement	Treatment^b^	Admission (A)	End of Month 1	End of Month 2	End of Month 3	End of month 4	*SED*^c^	Placebo^d^	Maca	*SED*	*P*^e^

BMI	APPMM	27.78	28.04	27.62	27.71	26.99	0.21*	27.83	27.47	0.16	<0.05
	AMMPP	27.38	27.59	27.33	27.11	27.48	0.21	27.29	27.46	0.16	ns
	*SED*		2.42	2.42	2.42	2.42		2.41	2.41		
	*P*		ns	ns	ns	Ns		ns	ns		
SBP	APPMM	118.5	125.7	118	120.5	110	5.1	121.8	117	4.2	ns
	AMMPP	120	123.5	113.5	111.5	114.4	5.1	112.9	118.5	4.2	ns
	*SED*		8.1	8.1	8.1	8.1		7.3	7.3		
	*P*		ns	ns	ns	ns		ns	ns		
DBP	APPMM	74.5	80.9	71	76	74	4.08	75.95	75.3	3.28	ns
	AMMPP	74	77	71	70	71.11	4.08	70.53	74.0	3.28	ns
	*SED*		5.94	5.94	5.94	5.94		5.25	5.25		
	*P*		ns	ns	ns	ns		ns	ns		

a A linear mixed model was fitted to data, allowing the comparisons between treatment groups: treatment by month differences in one model and Maca versus Placebo contrast in another model. The random effects included both individual variation and residuals. Two models may be written as follows: Month Model, Fixed (Treatment + Month + Interaction) + Random (Individuals + error); Treatment Model, Fixed (Treatment + Maca + Interaction) + Random (Individuals + error). The errors are assumed to follow a Gaussian (normal) distribution. All parameters were estimated using the Restricted Maximum Likelihood (REML) estimation. The differences between treatments within each month and between Maca and Placebo were tested using least significant difference (LSD) test at 5% and 1% significance levels;

b Average age of women in treatment sequence APPMM = 51.6 ± 1.29 and in AMMPP = 53.1 ± 0.75; Average time since the last period for treatment sequence APPMM = 12.6 and for AMMPP = 14.0 months;

c *SED*, Standard error of differences;

* existence of significant differences between monthly measurements within the same treatment sequence group. Values marked with unlike capital letters are considered statistically significant at *P*<0.05;

d Capital letters attached to the means indicate significant difference between values in columns. Small letters indicate significant differences between values in rows;

e *P*, Probabilities of significance. ns, not significant at *P*>0.05; <0.05, significance at 5% probability level; <0.01, significance at 1% probability level; <0.001, significance at 0.1% probability level.

Results from hormone assays summarized in Table 2 demonstrate that after two months of Placebo or Maca-GO treatment (prior to crossover), there were no significant differences existing between the two treatment sequences in FSH and PRG (*P*>0.05). However, Maca-GO significantly (*P*<0.05) increased overall E2 level with simultaneous significant (*P*<0.05) reduction of the overall LH levels as compared to Placebo treatment. The changes induced by Placebo treatment on E2 and LH prior to crossover into Maca-GO were more pronounced than the effect of Placebo introduced to participants after the Maca-GO treatment. This reduced effect of Placebo after previous Maca-GO application may indicate a residual effect of Maca-GO on women.

**Table 2:** Average Admission (A) and monthly values for FSH, E2, Progesterone (PRG) and E2 levels at five sampling points (Month Model)^a^ and Placebo (P) versus Maca-GO (M) contrast (Treatment Model) for two application sequences in a crossover design: A[PP × MM] and A[MM × PP] (n=11 in each group) during four months long study

Hormone	Treatment	Admission (A)	End of Month 1	End of Month 2	End of Month 3	End of month 4	*SED*^b^	Placebo	Maca	*SED*	*P*^c^

**FSH (mIU/ml)**	APPMM	64.62	70.54	71.28	65.6	60.66	8.08	70.91	63.56	5.6	ns
	AMMPP	69.03	62.01	57.27	53.63	59.22	7.465	56.42	59.64	5.29	ns
	*SED*		17.301	17.348	17.371	19.208		16.565	15.618		
	*P*		ns	ns				ns	ns		
**E2 (Pg/ml)**	APPMM	19.11	8.33	15.14	45.72	58.43	22.195	11.73	50.95	15.36	<0.05
	AMMPP	8.62	12.08	25.3	25.61	17.14	21.602	21.13	18.69	15.05	ns
	*SED*		31.661	28.375	29.016	35.96		18.992	19.124		
	*P*		ns	ns	ns	Ns		ns	ns		
**PRG (ng/ml)**	APPMM	0.201	0.203	0.213	0.277	0.286	0.232	0.208	0.282	0.176	ns
	AMMPP	0.224	0.233	0.373	0.281	0.252	0.281	0.266	0.303	0.125	ns
	*SED*		0.429	0.425	0.224	0.261		0.172	0.148		
	*P*		ns	ns	ns	ns		ns	ns		
**LH (mIU/ml)**	APPMM	43.79	38.92	38.91	34.05	32.41	3.985	38.91	33.38	2.74	<0.05
	AMMPP	36.66	32.69	32.58	30.03	32.5	3.678	31.27	32.64	2.59	ns
	*SED*		8.6	8.566	8.622	8.805		6.203	6.035		
	*P*		ns	ns	ns	Ns		ns	ns		

a For explanation see Table 1;

b *SED*, Standard error of differences;

c *P*, Probabilities of significance. ns, not significant at *P>0.05; <0.05*, significance at 5% probability level; *<0.01*, significance at 1% probability level; *<0.001*, significance at 0.1% probability level.

From the results of other hormone levels summarized in Table 3, it appears that there were no statistical differences (*P*>0.05) observed in concentrations of the TSH and T4 in blood of early-postmenopausal women while T3 and adrenal hormones (Cortisol and ACTH) showed statistically significant (*P*<0.05) reduction in recorded values as compared to overall Placebo effect in postmenopausal women. After the first month of Maca-GO application, in both sequence groups, T3 was noticeably increased but the second month of Maca-GO treatment resulted in substantial lowering of T3, below the admission and both months of Placebo treatment levels.

**Table 3:** Average Admission (A) and monthly values for TSH, T4, T3, Cortisol and ACTH levels at five sampling points (Month Model)^a^ and Placebo (P) versus Maca-GO (M) contrast (Treatment Model) for two application sequences in a crossover design: A[PP × MM] and A[MM × PP] (n=11 in each group) during four months long study

Hormone	Treatment	Admission (A)	End of Month 1	End of Month 2	End of Month 3	End of month 4	*SED*^b^	Placebo	Maca	*SED*	*P*^c^

TSH (μIU/ml)	APPMM	2.072	2.094	1.857	1.793	1.495	0.391	1.975	1.67	0.27	ns
	AMMPP	2.692	2.478	2.573	2.424	2.978	0.367	2.701	2.525	0.256	ns
	*SED*		0.817	0.817	0.817	0.84		0.774	0.779		
	*P*		ns	ns	ns	ns		ns	ns		
T4 (ng/100ml)	APPMM	1.177	1.183	1.081	1.159	1.194	0.037	1.177	1.132	0.029	ns
	AMMPP	1.21	1.169	1.1	1.216	1.09	0.039	1.135	1.164	0.031	ns
	*SED*		0.058	0.058	0.058	0.061		0.053	0.054		
	*P*		ns	ns	ns	ns		ns	ns		
T3 (pg/100ml)	APPMM	3.168	3.316	3.204	3.642	2.997	0.116	3.26	3.376	0.126	ns
	AMMPP	3.277	3.593	2.925	3.793	3.269	0.108	3.531	3.259	0.12	<0.05
	*SED*		0.218	0.218	0.218	0.225		0.216	0.22		
	*P*		ns	ns	ns	ns		ns	ns		
Cortisol (ng/ml)	APPMM	177.8	185.1	199.3	150.8	166.6	20.75	192.2	157.3	14.6	<0.05
	AMMPP	159.7	159.9	199.8	182.3	181	19.5	181.6	179.9	13.9	ns
	*SED*		26.3	26.3	26.3	28.1		22.4	22.8		
	*P*		ns	ns	ns	ns		ns	ns		
ACTH (pg/ml)	0PPMM	18.92	18.33	25.45	15.4	17.39	3.305	21.89	16.22	2.44	<0.05
	AMMPP	20	20.8	28.25	24.43	22.68	3.22	23.55	24.11	2.39	ns
	*SED*		5.56	5.56	5.56	5.79		5.14	5.24		
	*P*		ns	ns	ns	ns		ns	ns		

a For explanation see Table 1;

b *SED*, Standard error of differences;

c *P*, Probabilities of significance. ns, not significant at *P>0.05; <0.05*, significance at 5% probability level; *<0.01*, significance at 1% probability level; *<0.001*, significance at 0.1% probability level.

Table 4 which summarizes lipid and mineral metabolism data, shows that the overall effect of Maca-GO on Cholesterol, Triglycerides and HDL in relation to overall Placebo effect was statistically not significant (*P*>0.05). However, in relation to Placebo, Maca-GO treatment significantly increased (*P*<0.05) LDL with a simultaneous slight, but statistically not significant (*P*>0.05) increase in HDL contents as compared to sampling at the admission point.

**Table 4:** Average Admission (A) and monthly values for Lipids: Cholesterol (CHOL), Triglycerides (TRGL), High Density Lipoproteins (HDL) and Low Density Lipoproteins (LDL) and Minerals: Calcium (Ca), Phosphorus (P) and Iron (Fe) levels at five sampling points (Month Model)^a^ and Placebo (P) versus Maca-GO (M) contrast (Treatment Model) for two application sequences in a crossover design: A[PP × MM] and A[MM × PP] (n=11 in each group) during four months study

Measurement	Treatment	Admission (A)	End of Month 1	End of Month 2	End of Month 3	End of Month 4	*SED*^b^	Placebo	Maca	*SED*	*P*^c^

Lipids											
CHOL (mg/100ml)	APPMM	215.1	222.9	233.7	232.3	230.6	9.2	228.3	231.6	6.6	ns
	AMMPP	221.3	220.4	226.6	235	222.4	8.47	228.7	223.5	6.2	ns
	*SED*		21.1	21.1	21.2	21.7		20.2	20.2		
	*P*		ns	ns	ns	ns		ns	ns		
TRGL (mg/100ml)	APPMM	147.2	134.7	166.1	160.1	176.7	24.4	150.4	166.9	17.1	ns
	AMMPP	167	160.4	134.3	140.6	161.3	22.52	151	147.3	16.1	ns
	*SED*		40.7	40.7	41.1	42.9		37.8	37.9		
	*P*		ns	ns	ns	ns		ns	ns		
HDL (mg/100ml)	APPMM	59.41	66.73	67.72	67.82	70.07	3.19	67.23	68.75	2.27	ns
	AMMPP	64.66	64.77	66.2	68.29	61.25	2.94	64.77	65.49	2.15	ns
	*SED*		7.75	7.75	7.79	7.95		7.49	7.5		
	*P*		ns	ns	ns	ns		ns	ns		
LDL (mg/100ml)	APPMM	138	150.1	142.8	168	169.3	10	146.8	168.6	6.8	<0.01
	AMMPP	148.6	152.8	148	163.9	154.5	8.37	159.2	150.4	5.9	ns
	*SED*		20.7	21	21.2	21.6		20	20.1		
	*P*		ns	ns	ns	ns		ns	ns		
Minerals											
Ca (mEq/L)	APPMM	4.46	4.73	5.03	4.8	4.714	0.085	4.88	4.765	0.075	ns
	AMMPP	4.49	4.69 B	4.95 A	4.73 B	4.63 B	0.085*	4.68	4.82	0.071	ns
	*SED*		0.119	0.119	0.119	0.119		0.104	0.104		
	*P*		ns	ns	ns	ns		ns	ns		
P (mg/100ml)	APPMM	3.22	3	3.16	3.14	3.143	0.203	3.08	3.141	0.157	ns
	AMMPP	3.27	3.66	3.19	3.62	3.28	0.203	3.45	3.425	0.157	ns
	*SED*		0.258	0.258	0.258	0.258		0.217	0.217		
	*P*		<0.05	ns	ns	ns		ns	ns		
Fe (mcg/100ml)	APPMM	69.27	82.08	80.78	81.39	82.23	10.51	81.43	81.74	8.02	ns
	AMMPP	85.45	120.1	101.97	97.49	75.38	10.51	86.43	111.04	8.02	<0.01
	*SED*		11.32	11.32	11.32	11.32		8.63	8.63		
	*P*		<0.01	ns	ns	ns		ns	<0.01		

a For explanation see Table 1;

b *SED*, Standard error of differences;

* existence of significant differences between monthly measurements within the same treatment sequence group. Values marked with unlike capital letters are considered statistically significant at *P*<0.05.

c *P*, Probabilities of significance.ns, not significant at *P>0.05; <0.05*, significance at 5% probability level; *<0.01*, significance at 1% probability level; *<0.001*, significance at 0.1% probability level.

After two months of Maca-GO treatment in AMMPP sequence group, there was significant (*P*<0.05) increase in serum Iron content in relation to both admission point and the second month of Placebo introduced after the Maca-GO treatment. Similarly to Iron, in AMMPP group, plasma Calcium level was significantly higher (*P*<0.05) after the second month of Maca-GO treatment as compared to Placebo after the crossover point and at the start of the Trial. In sequence group APPMM, after second month of Placebo treatment, there was a difficult to explain increase in Calcium level, however difference between Placebo and Maca block of data was not statistically confirmed (*P*>0.05).

### Bone Density

Results of bone density markers presented in Table 5 demonstrate that after four-month intake of Placebo capsules, there was a reduction in individual and combined bone density values and computed score, while during the same period, participants who self-administered Maca-GO capsules during four months observation, recorded noticeable increase in their bone density measures in relation to the admission point and to Placebo treatment.

**Table 5:** Forearm bone density results (Trubecular, Cortical + Subcortical, Total Density (mg/10^3^ mm) and Total Density Score “T-Score”) and related concentrations of two blood hormones (FSH and E2) in early-postmenopausal women at the Admission and after four months administration of either Placebo (A-P-P-P-P; n = 4) or Maca-GO (A-M-M-M-M; n=5)

Detection Point	At Admission		After 4 months		At Admission		After 4 months	

Measurement & Group	Trabecular Density (mg/10^3^mm)				Cortical + Subcortical (mg/10^3^mm)			

Bone Density	Mean	*SE*^a^	Mean	*SE*	Mean	*SE*	Mean	*SE*
A-P-P-P-P	176.6	12.1	177.3	12.6	497.7	13.5	478.5	23.9
A-M-M-M-M	187.2	11.9	186.1	13.4	534.2	34.7	552.8	44.4
	Total Density (mg/10^3^mm)				Total Density “Z-Score”			
Total Density & Score	Mean	*SE*	Mean	*SE*	Mean	*SE*	Mean	*SE*
A-P-P-P-P	357.1	15.3	343.1	17.4	0.0	0.15	-0.23	0.19
A-M-M-M-M	379.1	21.5	388.0	25.5	0.36	0.32	0.52	0.38
Hormone	FSH (mIU/ml)				E2 (pg/ml)			
A-P-P-P-P	82.20	3.86	73.40	4.11	7.23	0.45	13.23	0.96
A-M-M-M-M	68.25	3.27	30.50	3.07	13.52	0.94	31.82	4.17

a *SE* (±), standard error of mean.

Due to low number of women participating in the bone density observation test, results were expressed as groups’ means and standard deviation only, since calculation of statistically-significant differences would lack credibility. Observed increase in bone density markers due to Maca-GO administration was accompanied by a substantial decrease in FSH (55%) and an increase in E2 values (135%), while in Placebo group, during the same length of the Trial, there was much reduced change observed in the same hormone values (11% decrease for FSH and 83% increase in E2).

### Kupperman’s Menopausal Index (KMI) and Greene’s Menopausal Score (GMS)

The objective in analysis of menopausal symptoms was to examine the effect of Maca-GO treatment administered over variable time frames (one or two months) intermittently with Placebo (at pre and post Maca-GO administration), on the alleviation of menopausal symptoms as subjectively assessed by early-postmenopausal women during personal interviews with gynecologists. In analysis of the answers provided by participants to the questionnaires according to Kupperman and Greene, the emphasis was put on those symptoms which had been indicated by women at admission as having the most pronounced effects on their life as they entered menopause Listed in descending order of priority, the following symptoms reflect the degree of discomfort attributed to their early-postmenopausal stages: hot flushes, nervousness, excessive sweating (profuse perspiration), interrupted sleep pattern, depression, general weakness, headache, joint pain, heart palpitations, loss of body balance and numbness in hands and/or legs-feet.

### Indices of Menopausal Discomfort according to Kupperman’s and Greene’s questionnaires

After one month of treatment with either Maca-GO or Placebo, both sequence groups responded in similar, statistically significant (*P*<0.001) manner showing reduction in total values of KMI and GMS, with Maca-GO having much more pronounced effect on sum of the individual symptoms than the same length of Placebo treatment (Table 6). The second month of Maca-GO treatment magnified the positive effect on alleviation of individual menopausal symptoms as measured by KMI only. Extending Placebo treatment into the second month resulted in reversing the positive effects observed in total KMI values after the first month of Placebo use. This was particularly distinctive in the APPMM sequence group and when the KMI was used. Differences between Placebo and Maca-GO treatments were more pronounced in the KMI as compared to GMS with the GMS values showing significant (*P*<0.01) effect of Placebo in one sequence group only (APPMM) while KMI recorded values in both sequence groups showed highly significant (*P*<0.001) difference between Placebo and Maca-GO treatment.

**Table 6:** Dynamics of overall monthly changes in total values from individual symptoms determined according to Kupperman’s Menopausal Index (KMI) and Greene’s Menopausal Score (GMS) recorded between Admission point (A), at four monthly sampling points (Month Model)^a^ and Placebo (P) versus Maca-GO (M) contrast (Treatment Model) according to two sequences of Maca-GO application (pre- and post Placebo) during four month of crossover Trial (n=22)

Treatment	Admission A	After Month 1 P	After Month 2 P	After Month 3 M	After Month 4 M	*SED*^b^	Placebo	Maca	*SED*	*P*^c^

Kupperman’s Menopausal Index (KMI)										
OPPMM	30.40	15.20	24.00	10.80	6.99	1.69	19.60	9.65	1.31	<0.001
OMMPP	29.64	13.09	9.45	14.88	19.58	1.52	17.21	11.27	1.30	<0.001
*SED*		3.52	3.52	3.53	3.59		3.47	3.47		
Greene’s Menopausal Score (GMS)										
OPPMM	25.80	10.90	8.60	8.90	3.30	1.55	9.75	6.10	1.44	<0.01
OMMPP	30.91	12.45	10.91	13.00	7.18	1.48	10.09	11.68	1.37	ns
*SED*		4.28	4.28	4.28	4.28		4.35	4.04		

a For explanation see Table 1;

b *SED*, Standard error of differences;

c *P*, Probabilities of significance. ns, not significant at *P>0.05; <0.05*, significance at 5% probability level; *<0.01*, significance at 1% probability level; *<0.001*, significance at 0.1% probability level.

When Placebo was introduced after previous Maca-GO period, then there was a statistically significant increase (*P*<0.001) in KMI values (Table 6) indicating an increase in severity of menopausal symptoms contributing to the computed KMI values after Maca-GO was withdrawn.

Since GMS was less sensitive to changes induced by both Maca-GO and Placebo, the severity of individual symptoms in relation to Maca-GO and Placebo were further assessed using KMI test only. Analyzing KMI results for individual 11 symptoms as recorded in the APPMM sequence treatment (Table 7), one month Placebo administration resulted in highly significant (*P*<0.001) reduction in severity of most of the menopausal symptoms with, excessive sweating, interrupted sleep pattern, nervousness, depression, loss of body balance, heart palpitations and numbness in hands & legs being most pronounced (*P*<0.001), with KMI values in remaining symptoms also significantly lowered, but to a lesser degree (*P*<0.05). With the second month of Placebo application, values of most KMI symptoms were increased, as compared to the first month values, with the exception of loss of body balance, joint pain and heart palpitations. On the other hand, KMI values for hot flushes and excessive sweating after the second month of Placebo treatment increased to a higher level than those recorded at the Admission point. Introducing Maca-GO after the second month of Placebo treatment significantly (*P*<0.001) reduced KMI values for the following symptoms: hot flushes, excessive sweating, interrupted sleep pattern and nervousness, while values for other symptoms were less (*P*<0.05) or not significantly (*P*>0.05) affected.

**Table 7:** Monthly changes in values of individual symptoms determined according to Kupperman’s Menopausal Index (KMI) recorded for APPMM treatment sequence (n=11) between Admission (A), and four sampling points at monthly intervals (Month Model)^a^ with two months Placebo (P) followed by two months Maca-GO (M)

Symptom as per KMI	Admission A	After 1m P	After 2m P	After 1m M	After 2m M	*SED* (±)^b^	*P*^c^	Placebo	Maca	*SED*^b^	*P*^c^

Hot flushes	2.20	1.70	2.60	1.00	0.83	0.251	<0.001	2.15	0.92	0.17	<0.001
Excessive sweating	2.10	0.70	2.20	1.10	0.71	0.396	<0.001	1.45	0.91	0.29	0.066
Interrupted sleep	2.30	1.20	1.80	0.60	0.57	0.399	<0.001	1.50	0.59	0.29	0.004
Nervousness	2.50	1.00	1.90	0.30	0.14	0.262	<0.001	1.45	0.22	0.18	<0.001
Depression	1.30	0.40	0.20	0.50	0.43	0.354	0.034	0.30	0.46	0.20	0.407
Balance	0.90	0.10	0.10	0.20	0.00	0.199	<0.001	0.10	0.10	0.10	1.000
General weakness	1.90	1.10	0.60	1.00	0.14	0.397	0.001	0.85	0.57	0.28	0.318
Joint pain	1.20	0.40	0.40	0.60	0.25	0.375	0.115	0.40	0.43	0.23	0.912
Headaches	1.00	0.40	0.10	0.20	0.14	0.237	0.002	0.25	0.17	0.13	0.563
Heart palpitations	1.10	0.20	0.20	0.20	0.14	0.263	0.003	0.20	0.17	0.13	0.832
Numbness hands & legs	0.40	0.00	0.20	0.10	0.14	0.166	0.189	0.10	0.12	0.10	0.836
Total value for KMI symptoms	16.9	7.20	10.30	5.80	3.51	0.185	<0.001	19.60	9.40	1.31	<0.001

a For explanation see Table 1;

b *SED*, Standard error of differences;

c *P*, Probabilities of significance. ns, not significant at *P*>*0.05*; <*0.05*, significance at 5% probability level; <*0.01*, significance at 1% probability level; <*0.001*, significance at 0.1% probability level. Scoring index: 0, symptom not experienced; 1, occasionally; 2, often; 3, very often; Kupperman’s Indexing Factors: for K-1, ×4; K-2 to K-5, ×2; the remaining, ×1).

### Canonical variate analysis of results derived from Kupperman’s Index and Greene’s Score

**Symptoms according to Kupperman’s Menopausal Index**: Vector loadings (Table 8) of the first canonical variate for all the treatment groups in this and previous part of the study (1) shows that at the Admission point, general weakness was the symptom with the largest coefficient value (coefficient loading) of the first canonical variate equation. This indicates that this symptom was the most important indicator of post-menopause separating treatment groups. This was followed by nervousness, excessive sweating and interrupted sleep pattern. Hot flushes were the symptom with the largest coefficient loading for the 2^nd^ canonical variate equation, followed by interrupted sleep pattern and depression. It is important to notice that hot flushes were statistically highly correlated (r=0.315; *P*<0.01) with the excessive sweating, which may be one of the reasons that the symptom excessive sweating was not so important in the equation, because hot flushes have been a dominating symptom as per outcome of the analysis for the 2^nd^ canonical equation.

**Table 8:** Coefficients (vector loadings) of the first and second canonical variate equations for Kupperman’s Menopausal Index as calculated for the Admission point and after one, two, three and four months of Maca-GO treatment sequences applied intermittently with Placebo using the data from the previous part (1) and the present part of the study (Total n=146 women)

Symptom (Kupperman’s Index)	Coefficients (vector loadings) of 1^st^ canonical variate equations					Coefficients (vector loadings) of 2^nd^ canonical variate equations				
	Month 0A	Month 1	Month 2	Month 3	Month 4	Month 0A	Month 1	Month 2	Month 3	Month 4

Hot flushes	0.040	-0.001	-1.101	0.728	-2.561	1.016	1.035	-0.297	-1.448	0.114
Excessive sweating	0.359	-0.303	-0.055	0.531	0.227	-0.318	-0.549	0.202	0.193	0.137
Interrupted sleep pattern	0.353	-0.253	-0.658	0.033	-0.341	0.438	0.443	0.049	0.254	1.131
Nervousness	0.614	-0.834	-0.432	0.198	-0.271	-0.377	-0.050	0.181	0.309	-0.174
Depression	-0.419	-0.066	-0.114	0.104	-0.169	0.388	0.467	0.679	-0.161	0.350
Losing body balance	-0.332	0.056	0.505	0.562	-0.187	-0.534	-0.847	-0.348	0.362	0.229
General weakness	0.945	-0.536	0.025	0.518	0.423	-0.168	0.414	0.578	0.728	-0.848
Joint pain	0.147	-0.453	-0.072	0.315	0.234	-0.098	-0.651	0.564	0.107	1.729
Headaches	-0.240	0.089	0.282	-0.457	-0.420	-0.016	-0.331	0.066	-0.267	-0.115
Heart palpitations	-0.153	0.088	-0.033	-0.370	1.117	-0.306	-0.502	0.016	0.633	-0.120
Parestesy/Numbness	-0.003	0.115	-0.038	-0.540	-0.165	-0.418	-0.013	0.181	-0.905	-0.339

After 2 months administration of Maca-GO or Placebo treatment, hot flushes showed the highest coefficient (in absolute value) in the first canonical variate equation (Table 8), which indicated statistically significant separation of the treatment groups (Placebo from Maca-GO), followed by such symptoms as interrupted sleep pattern, nervousness and loss of body balance. In the 2^nd^ canonical equation such symptoms as depression, general weakness and joint pain were the top 3 factors separating the treatment groups. The correlation between hot flushes and excessive sweating increased after 2 months of treatment. After the second month, hot flushes were also significantly correlated with interrupted sleep pattern (*P*<0.01) and nervousness (*P*<0.05).

After the fourth month of the study, hot flushes remained the highest coefficient of the 1^st^ canonical equation, followed by heart palpitation (Table 8). In the 2^nd^ canonical equation symptoms of joint pain, interrupted sleep pattern and general weakness, were mostly influencing the treatment group separation. At this stage hot flushes were no longer related to excessive sweating, interrupted sleep pattern or nervousness, which may support observations that Maca-GO significantly reduced hot flashes as a prime symptom affecting early-postmenopausal women at the time of admission to the study.

**Figure 1:**
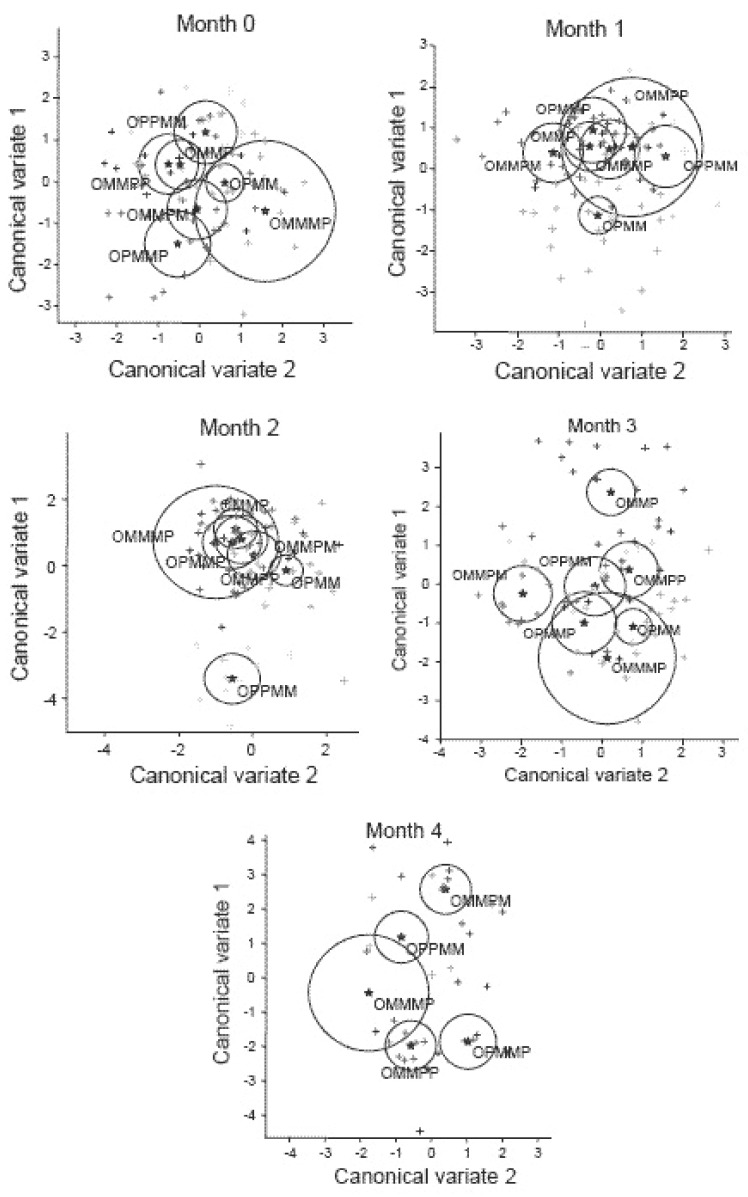
Canonical scores of 1st and 2nd variate transformed from Kupperman’s defined individual postmenopausal symptoms (KMI) for Months 0 = Admission Point and after monthly (1 to 4) intake of Maca-GO capsules according to treatment sequence applied intermittently with Placebo and computed using the data from the previous part (1) and the present part of the study. *(Data for treatment sequences within each of the computed encircled clusters displayed as the vectors of combined 11 KMI symptoms, when separated from each other, shows significant difference at the *P*<0.05 level).*

Figure 1 shows separation of the individual treatment groups by canonical variate analysis of KMI prior to (Month 0) and after the 1^st^, 2^nd^, 3^rd^ and 4^th^ months of application of the individual sequences of treatments in the whole study (for all the sequence groups used in previous part (1) and this trial. Group sequence APPMM was segregated from the rest of groups by the first canonical variate. Group APMM was separated from AMMPM and APPMM by the first canonical variate after one month of study, while Group AMMPM was separated from APPMM by the second canonical variate. Two months application of all treatment sequences to women resulted in a separation of Group sequence APPMM and APMM from the remaining treatments. After four months of applying treatment sequences, all the four Groups: AMMPP, APPMM, AMMPM and APMMP were discriminated by the canonical variates. Figure 1 also shows that the participants from the three groups (APMM, AMMP and APPM) were clustered separately by the canonical variate analysis even before the treatments were administered.

**Figure 2:**
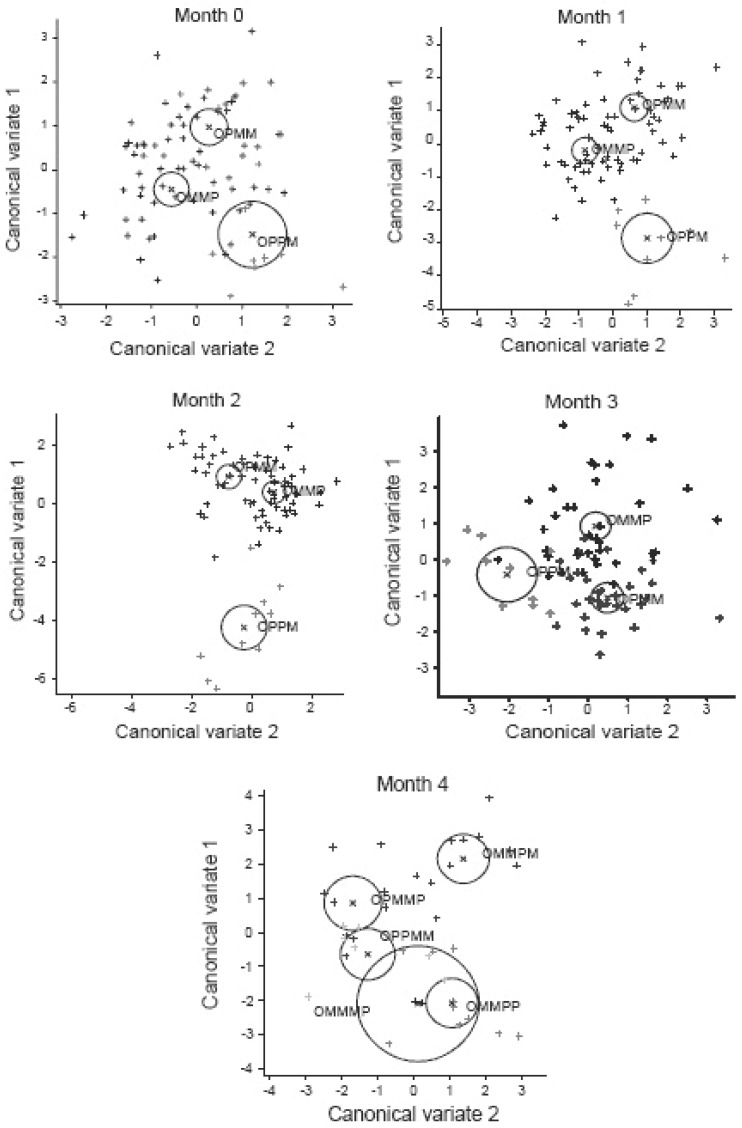
Canonical scores of 1st and 2nd variate transformed from Greene’s defined individual menopausal symptoms (GMS) for months 0 = Admission Point and after monthly (1 to 4) intake of Maca-GO capsules according to treatment sequence applied intermittently with Placebo and computed using the data from the previous part (1) and the present part of the study. (Data for treatment sequences within each of the computed encircled clusters displayed as the vectors of combined 21 GMS symptoms, when separated from each other, shows significant difference at the *P*<0.05 level).

**Symptoms according to Greene’s Menopausal Score:** Using scores recorded for individual symptoms of the GMS for all the sequence groups used in this Trial and the previous part of the study (1) and relevant vector loadings for the first and second canonical variate equations as determined and summarized in Table 9, the following individual GMS symptoms used as variables, were the most important indicators of postmenopausal conditions at the Admission to the study: nervousness, excessive crying, excessive alertness, sudden feeling of anxiety, excessive night sweating, headaches and irritability, nervousness and depression being of the highest importance at 1^st^ canonical variate and irritability, nervousness, unhappiness and depression were important symptoms at 2^nd^ canonical variate, which separated results collected in four sequence groups at Admission point. In order to compare separation of clusters during four months of study, in Figure 2 the overall degree of separation was demonstrated using clusters of GMS data for all the sequence groups as observed at Admission point (Month 0) and during four months of the relevant treatment sequences as used in the entire study. Similar pattern of separation was observed as the one obtained in analysis of KMI data shown in Figure 1, indicating separation of computed clusters of canonical scores for matrices of 21 menopausal symptoms and demonstrating that Maca-GO affected position of clusters represented by individual groups and significantly influenced reduction in severity of symptoms associated with early-postmenopause.

**Table 9:** Coefficients (vector loadings) of 1st and 2nd canonical variate equations defined by symptoms according to Green’s Menopausal Score (GMS) on early-postmenopausal women computed for Maca-GO treatment sequences applied intermittently with Placebo using the GMS data from the previous part (1) and the present part of the study (Total n=146 women)

Symptom	Coefficients of 1^st^ canonical variate equations from GMS					Coefficients of 2^nd^ canonical variate equations from GMS				
	Month 0	Month 1	Month 2	Month 3	Month 4	Month 0	Month 1	Month 2	Month 3	Month 4

Abnormally-fast heart rate	-0.320	-1.612	-1.526	0.810	-0.649	0.031	0.274	-0.152	0.216	0.719
Nervousness	-1.214	0.019	0.411	0.978	-0.706	0.589	0.441	0.178	0.646	0.072
Difficulty falling asleep	0.180	0.285	-0.150	0.016	0.648	0.361	0.265	-0.136	-0.150	-0.395
Excessive alertness	0.903	0.376	0.475	-0.608	0.293	-0.252	-0.239	0.041	-0.037	0.269
Sudden feeling of anxiety	0.767	0.444	-0.028	-0.658	-3.456	-0.248	1.263	-0.377	0.575	1.353
Difficulty concentrating	-0.026	0.338	0.406	0.381	-0.054	0.135	0.246	-0.677	-0.128	1.004
Feeling of tiredness/lack of energy	-0.110	-0.106	0.250	-0.023	-0.154	-0.277	0.177	0.159	0.009	0.725
Lack of interest	-0.248	0.223	0.016	-0.456	-0.825	-0.052	0.040	-0.024	0.451	1.661
Unhappy/depressed	-0.027	0.033	-0.109	0.272	0.483	0.549	0.083	-0.895	-0.455	-0.314
Excessive crying	-0.926	-0.346	0.010	0.572	2.124	0.159	-1.050	0.773	0.432	0.842
Irritability	-0.375	0.119	0.331	-0.460	1.476	-0.819	0.145	-0.802	-0.161	-1.578
Loss of consciousness	-0.198	0.552	0.252	0.698	0.466	-0.497	-0.609	0.514	0.668	-1.237
Nervous tension	0.274	0.480	0.477	0.323	1.112	-0.227	-0.332	0.786	0.249	2.038
Numbness / “pins & needles”	-0.202	-0.180	-0.027	-0.720	0.434	-0.162	0.122	0.499	0.271	-1.574
Headaches	0.357	-0.078	0.312	-0.056	-0.506	-0.339	-0.327	-0.038	0.542	1.443
Muscle and joint aches and pains	0.146	0.158	-0.095	0.376	-0.554	-0.042	0.243	-0.326	-0.107	-1.117
Loss of feeling in feet & hands	-0.043	-0.170	-0.037	-0.040	0.379	0.283	0.037	0.007	0.135	2.164
Difficulty breathing	0.283	0.363	0.534	-1.112	6.352	0.397	0.243	0.592	1.414	-1.839
Hot flushes	0.165	0.500	0.049	0.104	-1.696	0.249	0.540	-0.716	-0.287	-1.244
Excessive night sweating	-0.612	-0.144	-0.316	-0.049	0.912	-0.204	-0.283	-0.188	-0.324	0.156
Loss of interest in sex life	-0.086	0.175	-0.383	0.466	-0.340	-0.308	-0.287	0.579	-0.995	1.365

## DISCUSSION

### Hormonal balance after Maca-GO treatment in early-postmenopausal women

In the present study, applying Maca-GO treatment to early-postmenopausal women, in addition to a significant increase in E2, lowered LH, T3, CT, ACTH and steady FSH and PRG levels, there was a highly significantly reduction in BMI and both frequency and intensity of menopausal symptoms such as hot flushes, profound perspiration (night sweating), as well as reduced depression, irritability, difficulty in falling asleep and other as demonstrated by the KMI and GMS, which may indicate similar effect as the one induced by the HRT treatment. In addition, unlike the reported negative consequences of the HRT programs (11), Maca-GO has not contributed to an increase in blood pressure, nor triggered an increase in body weight, depression and mood swings, which all are considered as negative side-effects of HRT (11-14). Demonstrated in this study was the positive effect of Maca-GO on lowering E2, and a significant reduction in the range of menopausal symptoms not restricted to hot flushes and night sweating, with a simultaneous lowering in BMI without affecting blood pressure, together showing that Maca-GO has all the pre-requisites to become a natural non-hormonal treatment superior to HRT in terms of all the additional benefits not delivered by HRT programs.

A similar pattern in responses of women observed in FSH and LH results after application of Maca-GO in various sequence groups in the previous part of the study (1) with simultaneous reverse effects recorded in E2 values being significant in APMMP and APPMM sequence groups only (*P*<0.01 and *P*<0.05 respectively).The above results confirmed the trend observed in the previous part of the study (1) in which Maca-GO increased E2 and lowered FSH levels, while in the present Trial, E2 level only was increased without significant effect on FSH.

The existence of a positive relationship between Maca-GO treatment and its hormone balancing function in early-postmenopausal women observed in this study may be supported also by previous pilot observations on early-postmenopausal women (2) and in-depth biochemical and physiological observations made in bioassays using sexually-experienced (4) and ovariectomised laboratory animals (3).

### Maca-GO and thyroid function

According to Lucille (15), on the basis of his clinical experience, the balance between progesterone, estradiol and thyroid function is one of the key factors in the female, responsible for maintaining hormonal balance during the reproductive years and at menopause. It is a key function of progesterone to control estradiol and prevent the negative effects of its dominance during the pre-menopausal years as well as to support thyroid function in maintaining growth, healthy bone metabolism and to maintain psychological equilibrium in females during and after their reproductive stage of life. Such a relationship has been confirmed in this study, since Maca-GO treatment resulted in lowering T3 and elevation of E2, with positive effect on bone density markers and alleviation of psychological type menopausal symptoms in postmenopausal women enrolled in the study.

### Placebo effect on the outcome of Maca-GO treatment

There was a significant response of early-postmenopausal women in hormonal profiles and menopausal symptoms after one month Placebo treatment, introduced immediately after the admission to the Trial. This effect was eliminated or substantially reduced after the second month of Placebo treatment, which indicates an existence of a distinctive Placebo effect, which should be taken into account in observation of results from treatments on postmenopausal women. This observation is consistent with previous reports (1) on use of the Maca-GO where there was a strong indication of the existence of Placebo effect linked to emotional response of participant, in expectation of a positive effect of “the treatment” to which they were exposed.

**Figure 3:**
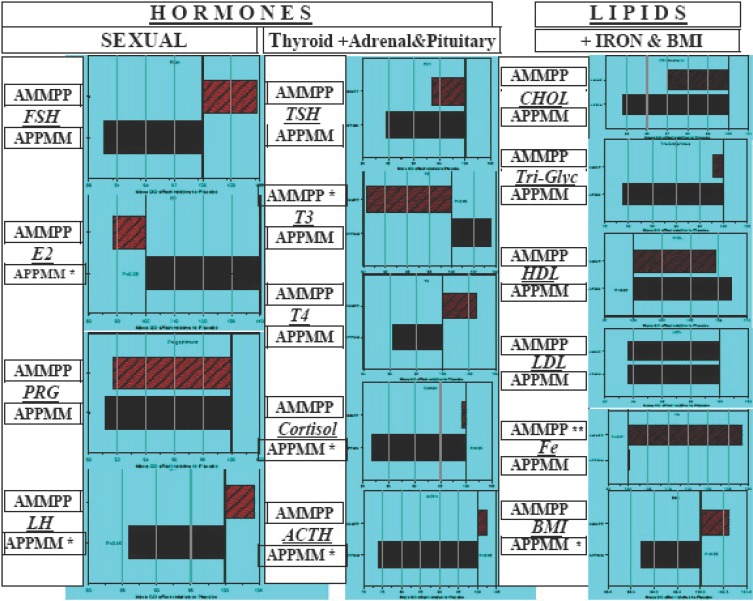
Comparison of Placebo-corrected values for hormones: FSH (IU/ml), E2 (pg/ml), PRG (ng/ml) and LH (mU/ml), TSH (μIU/ml), T3 (pg/ml), T4 (pg/ml), Cortisol (μg/100ml) and ACTH (pg/ml), lipids: CHOL (mg/ml), Triglycerides (mg/100ml), HDL (mg/ml) and LDL (mg/ml), Iron (mcg/100ml)and BMI, after two months Maca-GO treatment (relative to Placebo) demonstrating the effect of two month Placebo when introduced prior to, or after Maca-GO capsules were administered to early-postmenopausal women according to APPMM or AMMPP treatment sequence.

This study also confirmed the existence of a residual effect of Maca-GO, clearly visible in the series of measurements recorded and summarized in Figure 3. Analytically determined results have full support in trends recorded as subjective responses of women to GMS and KMI determined at the time of blood sampling for analyses of hormones and other blood constituents. The majority of women, who after Placebo run-in period received Maca-GO capsules, recorded highly significant (*P*<0.001) reduction in feeling of menopausal discomfort as expressed by the total point scores of the GMS and KMI (Table 6). On the other hand women who changed from Maca-GO to Placebo treatment observed gradual, but not significant (*P*>0.05) increase in feeling of menopausal discomfort, with severity of symptoms increasing with the duration of the Placebo treatment.

It was also apparent from results in this study that the length of Maca-GO treatment had an effect on most of the measurements taken, as based on comparison of results recorded after one or two month of Maca-GO application, with two months of application magnifying the therapeutic effects which are observed after the first month of Maca-GO use. Similar trends were observed in GMS and KMI total values (Table 6), as well as in the example of individual menopausal symptoms summarized in Table 7, clearly demonstrating, that one month of Maca-GO treatment was not sufficiently long to exhibit full therapeutic effects on individual menopausal symptoms experienced by early-postmenopausal women. The above results are consistent with observations reported in the previous part of this study (1), in a pilot study on early-postmenopausal women (6) and in bio-assays on adult female and male rats (4).

### Maca-GO dosage - quantitative considerations

Depending on circumstances, which are almost always dependent on the dosage involved, Maca may exhibit a “stimulating” or a “balancing” effect on the organism. Based on clinical experience, reported by Muller (14), most women with menopausal symptoms, need a minimum of three to four 500 mg capsules with Maca powder daily, therefore in this study, four 500 mg capsules were used as arbitrarily-chosen daily dose 2 g per each participant. It is however important to note, that the very sensitive menopausal woman may need only two 500mg capsules per day (14). Dosage can be increased on a weekly basis and, if this amount is not sufficient, until the optimum (minimum effective) dosage is found. Taken at the correct dosage, which differs for each woman, Maca-GO may be able to reduce or completely eliminate hot flashes in women, in as little as 4 days to a week (14). It is also important to mention that, if the dosage is too high - and some women are very sensitive - the Maca will have a stimulating and not balancing effect, and will actually increase the number of hot flashes. If this happens, Maca-GO users can cut the dosage in half for a week, and then re-evaluate. If the problem persists, cutting the dosage in half again may help to identify the optimal daily dose (14).

It is therefore recommended for women using Maca-GO on their own, without a health practitioner’s supervision, to start with a small dose of the product and gradually increase its intake, as needed. However, in order to find precisely, the optimal dose of Maca-GO, which is sufficient and most appropriate to the particular physiological status of the woman, it is advisable to consult a health practitioner. He can order laboratory tests to establish base line hormone levels before starting the Maca-GO therapy, with a follow up, second series of hormone tests some two months later, after the initial daily dose of Maca-GO has been introduced, so as to establish the dose most appropriate to individually-adjusted physiological optimum for assisting in alleviation of menopausal symptoms.

Observations made in this and the previous study on Maca-GO (2, 4) as well as results reported elsewhere (14), shows that potential “stimulating” or “balancing” action of Maca as an adaptogen, is dependent on the level and the length of Maca intake resulting in a display of different therapeutic end-effects. Therefore, in the further study, it will be essential to establish a range of quantitative administrations of Maca-GO which would act within acceptable degrees of certainty, either as a stimulating or balancing dietary supplement, custom-designed for well-defined groups of subjects in their specific physiological state (gender, age, weight etc.) and again, for specific end-result in terms of the purpose and expected functionality.

### Maca-GO and its effect on blood Iron level

The positive effect of Maca-GO on Iron level in blood of early-postmenopausal women as observed in this study, is in accord with results obtained in a laboratory models on rats (3, 4) and reported in the literature (16), indicating that Maca-GO may play an important role in stimulation of absorption of dietary Iron from the digestive tract and possibly other minerals present in the diet. Although Maca-GO has not visibly affected levels of serum Calcium and Phosphorus in this study, the observed positive effect of Maca-GO treatment on bone density measures may indicate that Maca-GO may stimulate absorption of minerals from the digestive tract and their retention in the body by their deposition in bones, hence, their low levels in the circulating blood. It appears that Maca-GO may have different effects on the metabolic pathways relating to digestion and absorption of Calcium and Phosphorus from the effect observed in Iron, which was retained in blood circulation, assuming its priority of use in hemoglobin synthesis and red blood cells’ regeneration and formation.

### Maca-GO and its effect on mineral metabolism and bone density

**Figure 4:**
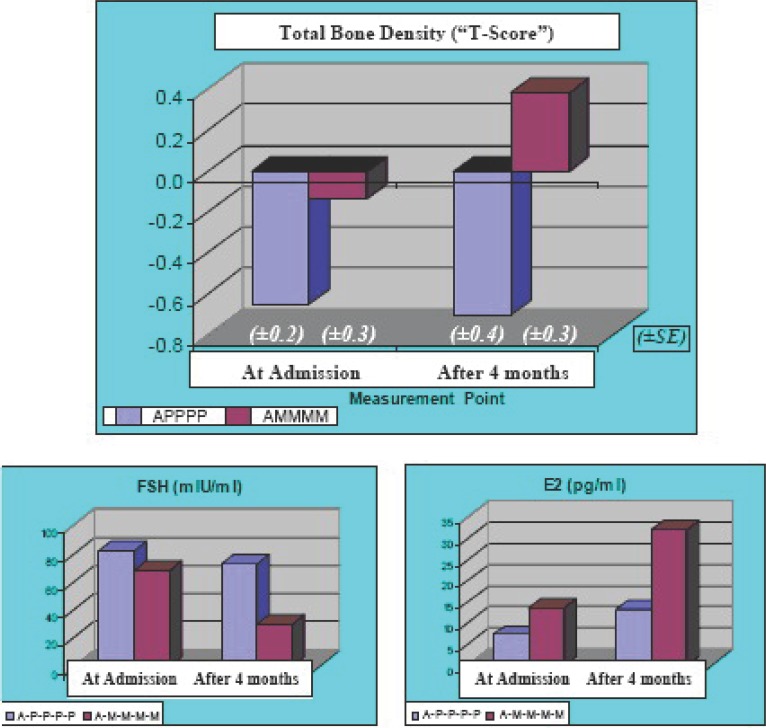
Forearm bone density results expressed as Total Bone Density Score (“T-Score”) and corresponding hormone concentrations (FSH and E2) in early-postmenopausal women, measured at the Admission Point (A) and after four months administration of two treatment sequences: either Placebo (A-P-P-P-P) or Maca-GO (A-M-M-M-M).

An important observation was made that after only four months of Maca-GO treatment, a distinctive increase in bone density (T-Score) was recorded (Figure 4), which confirmed earlier observations made in laboratory models, in which bones and muscles of test animals showed noticeable increase in Calcium and Phosphorus content (4). It may suggest that Maca-GO has a positive influence on absorption from the digestive tract of those two dietary minerals responsible for calcification of bones (and possibly other minerals not analyzed in this study), since the quantity of daily Calcium and Phosphorus intake present in the total 2 g Maca-GO in four capsules was only 6 mg - 7 mg, compared to approximately 1,000 mg daily Calcium supplementation needed to prevent bone density loss and retard the progress of osteoporosis in postmenopausal women (17). Increased bone density observed in this study in women receiving Maca GO treatment, supports an assumption that Maca-GO may stimulate absorption and/or re-absorption of Calcium and other key minerals from the diet, thus providing metabolic support in preventing loss of bone mass in postmenopausal women.

This loss of bone mass in turn, may lead to progression of, or development of osteoporosis and all the symptoms associated with it, including an increased excretion of Calcium in the urine which occurs with high intake of protein and sugars. Such impairment in bone calcification has been observed with high consumption of soft drinks, which is a common cultural- and socio-economic-related issue in the United States and other industrially-developed countries where osteoporosis and reduced calcium level in adults and children is becoming a major medical problem (18).

Irrespective of the dietary supplement(s) used, in order to prevent or reduce loss of bone density by postmenopausal women, an adoption of the following dietary measures can help to achieve this goal: eating a medium protein, low sugar, low sodium and fiber-rich diet, reducing intake of high phosphate-containing soft drinks, including Coca-Cola and Pepsi (18), introducing low or medium intensity exercise into daily routine (aerobic, weight-building, Pilates) and having sufficient Omega 3 fats (including ground flax seeds or oil and fatty fish in the diet).

### The effect of Maca-GO on individual menopausal symptoms

It appears that in comparison to Placebo, women participating in this study responded to two months Maca-GO treatment by significantly increasing E2 level which was associated with alleviation of a number of individual menopausal symptoms, most distinctive being a reduction in frequency and severity of hot flushes, excessive sweating, interrupted sleep pattern, nervousness depression, headaches and loss of libido - interest in sex life (Table 7). Studies reported by other authors (19) also indicate that Maca can be helpful in reducing discomfort caused by menopausal symptoms. Calculated combined effect of Maca-GO on alleviation of individual menopausal symptoms in early-postmenopausal women expressed in the format of canonical variate equations depicted in Figure 1 for 11 symptoms according to KMI and for 21 symptoms according to GMS - Figure 2, confirm that irrespective of the test used, both frequency and severity of symptoms were significantly reduced. Extending the length of Maca-GO treatment from one to two months reduced this effect further. This was demonstrated by increased separation between clusters of values for menopausal symptoms recorded with the progress of the study and an extension of Maca-GO use from one to two months period.

Both menopausal tests demonstrated that Maca-GO may also be considered as a non-hormonal energizing supplement with significant sedative, calming and anti-depressive effects, helping to improve concentration and alertness, these facts confirming results obtained in earlier pilot study (2) and demonstrated in laboratory models using bioassays on ovariectomised rats (3).

Significant reduction in symptoms of depression (KMI; K-5) after two months of treatment with 2 g daily dose of Maca-GO was parallel with significant reduction in CT and ACTH in the AMMPP sequence group only, while in APPMM group, reduction in symptom of depression was apparent without reduction in CT and ACTH levels. In the previous study on laboratory animals (4), at both high and low levels of intake, Maca-GO significantly lowered CT, while ACTH was distinctively, although not significantly, increased at low dose and distinctively lowered at the high Maca-GO dose. This observation indicated that anti-depressive effect of Maca-GO treatment was a dose-related response during short-term administration. However, during an extended use of Maca-GO (90 days), both CT and ACTH were substantially lowered, which could be an indication of the positive effect of longer-term use of Maca-GO in reducing symptoms of depression frequently affecting menopausal women. Results obtained in the present study with Maca-GO reducing depressive symptoms, confirmed reports in the literature (21, 22) of the close association existing between depressive symptoms and elevated CT and ACTH levels. The same relationship as reported in the literature (20) was also observed in the earlier part of this paper on ovariectomised rats (3), which after 28 days Maca-GO administration showed antidepressive (Porsolt test) and sedative effect (locomotor activity test), associated with significant reduction in both CT and ACTH, - the relationship confirmed by results reported in this paper on postmenopausal women.

Responses of participants in alleviation of stress attributed to Maca-GO treatment, confirmed results of the previously conducted laboratory trials on rats (3, 4), where Maca-GO reduced blood CT, indicating possible positive effect of treatment on lowering susceptibility of rats to stress factors and sedative effect on laboratory animals - the existence of such relationship reported by Lopez Fondo et al. (23). The study conducted on the same samples of Maca-GO as used in this paper using laboratory model on rats (24), when tested against Fluoxetine, a known antidepressant agent, confirmed the assumption that Maca-GO possesses typical antidepressant-like characteristics. After Maca-GO administration to ovariectomised rats, both blood CT and ACTH as well as spontaneous activity and immobility time (Porsolt test) were significantly (*P*<0.05) reduced, while Fluoxetine induced an anti-depressive effect in control, non-ovariectomised animals only, without affecting ovariectomised rats. Fluoxetine increased the blood CT in non-ovariectomised rats only, without significantly affecting ACTH and spontaneous activity test values. This led to the conclusion that the anti-depressive action of Maca-GO is based on different modes of action in non- and ovariectomised rats as compared to the antidepressive effect of Fluoxetin, which can be translated to the expected different responses of pre- and post-menopausal women to Maca-GO. The above results from a model laboratory test (24) in conjunction with the results recorded in this study on postmenopausal women, suggest that active phyto-components present in Maca-GO act in a specific way to trigger release of body’s own steroids or by affecting the hypothalamus-pituitary-ovarian axis in women which results in triggering similar, but other than serotoninergic response mechanisms as induced by Fluoxetine, which, in turn results in its anti-depressive action demonstrated on rats (24). These anti-depressive and stress-reducing actions of Maca-GO would need further, more detailed study.

### Comparison of sensitivity of the GMS and KMI tests

Comparing the degree of statistical significance in influences of Maca-GO on KMI and GMI in terms of detected reduction in frequency and severity of menopausal symptoms, it appears that KMI is more sensitive in displaying the effect of both Placebo and Maca-GO treatments on the recorded scores of menopausal tests as compared to GMS (Table 6), the fact observed also in a previous part of the study on postmenopausal women (1).

### Energizing effect of Maca-GO

The model laboratory study with the use of Maca-GO in rats (4) resulted in a significant increase in blood glucose level, which may explain the energizing effect of Maca-GO on early-postmenopausal women as observed in this study. This observation may also indicate that Maca-GO may find its use as an energizing dietary supplement for sports people and those whose lifestyle requires energy reserves for intensive physical-related activity (12, 25, 26).

### Maca-GO and its adaptogen-like function

The most distinctive effect of Maca-GO, which doesn’t contain any phyto-hormones, was an increase in E2 at slight PRG elevation, parallel to a reduction in FSH concentration in early-postmenopausal women. This peculiar hormone balancing function displayed by Maca-GO prepared from powdered hypocotyls of *Lepidium peruvianum* Chacon and applied in its natural entire cohesive form to early-postmenopausal women, strongly supports the conclusion that it closely resembles functions attributed to an adaptogen. The full meaning of this currently adopted term is as follows: (i) It must cause only minimal disorders in the body’s physiological functions; (ii) It must increase the body’s resistance to adverse influences not by a specific action but by a wide range of physical, chemical, and biochemical factors; and (iii) It must have an overall normalizing effect, improving all kinds of conditions and aggravating none. Maca-GO conforms and is characterized by all three characteristics, which constitute the complex description of what is expected from an adaptogen in classification terms as has been accepted in relation to *Panax* ginseng (27). There may be several variations of those three characteristics but all have similar meaning in physiological and biochemical interpretation terms. In earlier years, terms “general tonic” (increasing the overall tone of the whole body) - or more specifically “adrenal tonic” (increasing the tone and function of the adrenal glands) were used but have been replaced by the modern term “adaptogen”, as more precisely-defined by the three characteristics as given above. There is a consensus of opinion that Maca exhibits strong biologic action characteristic to plant adaptogens, the opinion expressed by Muller (14), supporting observations based on results presented in this study.

Results obtained in this study, give reason to conclude that Maca-GO acted as a toner of hormonal processes along the axis Hypothalamus-Pituitary-Ovaries, significantly stimulating production of E2 without significant affect on PRG, with a simultaneous suppression of blood FSH, LH, T3, Cortisol and ACTH levels, together with an increase in blood Fe and bone density index, as well as alleviation of menopausal symptoms as per KMI and GMS and a decrease in BMI recorded after two months of Maca-GO treatment. It appears that Maca-GO, by exhibiting functions and properties characterizing adaptogens, offers an attractive addition to the choices available as non-hormonal plant alternative to HRT for balancing levels of gonadal, pituitary, thyroid and adrenal hormones and relieving symptoms of menopausal discomfort with hot flushes and night sweating in particular, thus providing early-postmenopausal women with an option to reduce dependence on HRT programs.

### Maca-GO as a non-hormonal plant preparation - possible mode of therapeutic action

From research conducted so far on composition of Maca hypocotyls and various powdered root preparations, it appears that it does not contain any analytically-determined plant estrogens, or hormones (28-31). It has been initially suggested (12), that action of Maca relies on plant sterols, acting as chemicals which trigger chain of biochemical reactions helping the body itself to produce or modulate production of hormones and other compounds, appropriate to the age and gender of the person taking it. In this respect, sterols in Maca may be used by the body with the help of the pituitary to improve adrenal and ovarian (or testicular) functions, and therefore affect the thyroid, the pancreas, and the pineal gland (which also makes melatonin and which may have some connection to improved quality of sleep as observed in this study). The above may be one of the possible explanations why Maca-GO is so much more effective than phyto-estrogens in regulating hormonal balance and mobilizing action of the endocrine glands to work better. The multi-functional effects of Maca on endocrine relationships may also explain reports in the literature, of its positive influence on stimulation of endocrine glands in regulating hormonal balances in the body (12) and particularly in women who have already entered the postmenopausal stage of life.

Maca exhibits specific, yet not fully understood endocrine effects, ranging from being an energizing plant (12), stimulating reproductive functions (2, 3, 4) and balancing hormones (2, 5, 6) as well as alleviating physical, physiological and psychological discomfort associated with menopause in women (5, 13, 14). Since individual active compound(s) which could be biochemically identified as the key active Maca root component(s) responsible for specific therapeutic functionality of Maca root, have not been yet clearly determined, the authors are referring to Maca-GO root in its entirety and cohesive complexity considered as a therapeutic unaltered herb with its historically-acknowledged and “traditionally-unquestioned” dietary and medicinal properties (5, 6, 12). It is reasonable to suppose that the complexity and uniqueness of components present in Maca root such as sterols (campesterol, stigmasterol and beta-sitosterol), polyunsaturated acids and their amides, called “macaenes” and “macamides” (32), aromatic glucosinolates (29-31, 33) and several alkaloids and others constituents of Maca - yet to be characterized, through their complex synergistic and/or interactive action, will eventually one day provide an answer to specific physiological action of standardized Maca preparations, applied at specific, individually-adjusted doses to be recommended for prophylactic and/or specific therapeutic effects for men and women.

According to Dini (28), reported in the literature, the aphrodisiac powers of Maca for men and women, may be ascribed to the presence of prostaglandins and sterols in the hypocotyls of Maca and overall fertility enhancing properties may be attributed to the presence of biologically-active aromatic isothiocyanates derived by hydrolysis of the glucosinolates and specifically due to benzyl isothiocyanate and p-methoxybenzyl isothiocyanates (29, 30). In addition, benzyl isothiocyanate present in Maca root has been reported to be a potent cancer inhibitor of mammary gland and stomach (31).

Remembering that Maca-GO itself, does not contain any hormones (2, 12-14, 28), the action of Maca hypocotyls, traditionally is linked to its coherent and unique complexity of integral active constituents such as alkaloids, sterols, glucosinolates, amino acids, fatty acids, minerals and others yet to be established (8, 29, 30). Selectively extracted groups of components such as macamides and macaenes (32) may remove synergistically-essential constituents from Maca roots, eliminating other biologically-active compounds from Maca, traditionally used by natives of Peru as a whole root. In such a form Maca exerts its expected (and observed for centuries), therapeutic action on the body by stimulating the pituitary to produce and secrete the precursor hormones, which in tern, elevate estrogen, and testosterone levels, with simultaneous help in balancing the adrenal glands, the thyroid and the pancreas. Therefore, it is reasonable to suppose that Maca-GO may regulate ovarian function rather than stimulating the ovaries as is the case when other phytoestrogenic preparations, such as black cohosh, soy, red clover and others are used.

### Inconclusive effect of Maca-GO on indices of lipid metabolism

Observed in this study, the significant increase (*P*<0.05) in LDL concurrent with a stable level of HDL after two months Maca-GO application in relation to Placebo, was inconsistent with previously obtained results (1) showing Maca-GO significantly increasing HDL only, without affecting LDL, CHOL, TRGL. While, the previous results (1) showed a distinctive reduction in LDL/HDL ratio (from 2.7 to 2.3) after Maca-GO treatment, then, similar ratio in this Trial was minimally increased (from 2.2 to 2.4), but maintained at the levels similar in magnitude to the one observed after Maca-GO application in the previous part of the study (1). Lack of consistency in HDL and LDL results obtained in this and the previous study (1) on early-postmenopausal women, together with data in the previous laboratory study on adult (4) and ovariectomised rats (3), may suggest that the reports in the literature of hypo-lipidemic effect of Maca (33) may be dependent on a number of factors such as genetics, dietary habits, lifestyle, exercise, physiological constitution, ethnic predispositions (34), anti-oxidative properties (35) and environmental circumstances, etc. This aspect of lipid metabolism in relation to Maca-GO needs further consideration in view of the observation in this study of significant decrease in BMI after two months Maca-GO treatment, which may indicate its positive effect on reducing weight at the expense of fat tissues - a very desirable outcome of treatment for women in their pre- and postmenopausal stage, and specifically for those women opting for non-hormonal approach - as an alternative to HRT at early stages of their hormonal transition time.

### Closing remarks

Only one level of Maca-GO (2 g/day) was used in this clinical study, without information recorded on the dietary habits of participants, level of physical exercise, socio-economic and environmental considerations etc., which may appear, from the data reported in this and previous part (1) of the study, to be important factors in personalizing wellbeing of women during and shortly after menopausal transition. The complex mode of action of Maca is still far from fully understood. Therefore, in view of the demonstrated multi-factorial metabolic action of *Lepidium peruvianum* Chacon as an important cultivated therapeutic Andean plant used as dietary supplement (36), it would be of further practical and academic interest to investigate gender-related Maca-GO issues in relation to dosage levels, therapeutic effect, availability of key dietary and active compounds influencing menopausal women, dietary habits, body mass, exercise, geographical location, socio-economic status, including habitual and environmental considerations and other.

Results of the present study and previously reported work (2-4, 7), demonstrate that Maca-GO exhibits all the characteristics of a non-hormonal therapeutic preparation with functions ascribed to an adaptogen. This indicates that through balancing effects on the body, Maca-GO helps to produce its own optimal hormonal equilibrium, thus, providing a natural alternative in preventing and/or helping to treat hormonal dysfunctions or imbalances in early-postmenopausal women. This hormone-balancing function of Maca-GO appears to be a much more appropriate proposition, as an alternative to the generally-practiced routine of supplying women with synthetic, semi-synthetic or “bio-identical hormones” from outside sources or phyto-estrogenic preparations, as is the case in various hormone therapy and HRT programs.

## CONCLUSIONS

Maca-GO applied in parallel with Placebo during four months cross-over Trial, significantly increased E2, LDL and Iron (*P*<0.05), reduced FSH, T3, Cortisol, ACTH, LH, BMI (*P*<0.05) and noticeably improved bone density markers.

Maca-GO treatment significantly (*P*<0.001) lowered total KMI and GMS, relieving symptoms responsible for negative physiological and psychological manifestations, frequency and severity of flushes and night sweating - in particular, which are recognized under commonly-used term of “menopausal discomfort”.

In addition to hot flashes and profuse sweating, Maca-GO treatment significantly (*P*<0.01) alleviated such menopausal symptoms as, nervousness, mood swings, interrupted sleep pattern, fatigue, stress, headaches depression, and decreased libido observed in early-postmenopausal women.

Computed canonical variate equations for 11 or 21 symptoms according to KMI and GMS respectively, confirmed significant progressive reduction in both frequency and severity of symptoms with the length of Maca-GO treatment from one to two months.

After two month treatment, the 1^st^ and the 2^nd^ canonical vector loadings for 11 KMI symptoms showed significant (*P*<0.01) energizing effect of Maca-GO in early-postmenopausal women, with a significant improvement in concentration accompanied by an antidepressant-like and sedative influence.

For Maca-GO to exhibit its significant hormone-balancing and therapeutic effect, it was essential to use it continuously during two consecutive months.

After Maca-GO treatment was replaced for one month by Placebo, there was a reduced degree of Placebo effect on women as compared to Placebo introduced prior to Maca-GO treatment which indicates an existence of “residual effect” of the treatment.

Observed in this study were multi-functional actions and multi-directional effects of non-hormonal Maca-GO administration on endocrine relationships, which may explain its positive influence on endocrine glands to regulate the hormonal balance in women, who have just entered menopause.
